# Sulfur Mustard Toxicity Following Dermal Exposure: Role of Oxidative Stress, and Antioxidant Therapy

**Published:** 2007-10-30

**Authors:** Victor Paromov, Zacharias Suntres, Milton Smith, William L. Stone

**Affiliations:** Department of Pediatrics, East Tennessee State University, Johnson City; Northern Ontario School of Medicine, Advanced Technology and Academic Centre, 955 Oliver Road Thunder Bay, ON P7B 5E1; AMAOX, Ltd., #208, 6300 N. Wickham Rd, Melbourne, Fla

## Abstract

**Objective:** Sulfur mustard (bis-2-(chloroethyl) sulfide) is a chemical warfare agent (military code: HD) causing extensive skin injury. The mechanisms underlying HD-induced skin damage are not fully elucidated. This review will critically evaluate the evidence showing that oxidative stress is an important factor in HD skin toxicity. Oxidative stress results when the production of reactive oxygen (ROS) and/or reactive nitrogen oxide species (RNOS) exceeds the capacity of antioxidant defense mechanisms. **Methods:** This review will discuss the role of oxidative stress in the pathophysiology of HD skin toxicity in both in vivo and in vitro model systems with emphasis on the limitations of the various model systems. Evidence supporting the therapeutic potential of antioxidants and antioxidant liposomes will be evaluated. Antioxidant liposomes are effective vehicles for delivering both lipophilic (incorporated into the lipid bilayers) and water-soluble (encapsulated in the aqueous inner-spaces) antioxidants to skin. The molecular mechanisms interconnecting oxidative stress to HD skin toxicity are also detailed. **Results:** DNA repair and inflammation, in association with oxidative stress, induce intracellular events leading to apoptosis or to a programmable form of necrosis. The free radical, nitric oxide (NO), is of considerable interest with respect to the mechanisms of HD toxicity. NO signaling pathways are important in modulating inflammation, cell death, and wound healing in skin cells. **Conclusions:** Potential future directions are summarized with emphasis on a systems biology approach to studying sulfur mustard toxicity to skin as well as the newly emerging area of redox proteomics.

## SULFUR MUSTARD: A CENTURY OF THREAT

Sulfur mustard (SM) or mustard gas (bis-2-(chloroethyl) sulfide, military code: HD) is a chemical warfare agent classified as a weapon of mass destruction. Mustard gas was one of the first chemical weapons deployed against troops on a battlefield during World War I, almost hundred years ago. Since then, the military use of mustard gas has been documented in a number of situations. In 1988, HD was used with devastating results by Saddam Hussein's military forces against civilian targets in Halabja and later during the Iran-Iraq war. Mustard gas produces casualties in the battlefield and forces opposing troops to wear full protective equipment thus slowing the tempo of military operations. It is highly probable that mustard gas could be used by terrorists since it is a simple chemical compound readily synthesized without elaborate technology. Moreover, as a “persistent agent” (US Army classification) aerosolized mustard gas presents a threat for up to 1 week under dry and warm weather conditions because it remains in the environment until fully hydrolyzed. Along with nerve agents, mustard gas presents a major threat as a potential and effective chemical weapon. The possibility of low technology production, easy stockpiling, and difficulty in verifying its storage makes mustard gas a continuing worldwide threat. Presently, there is no antidote or effective treatment for mustard gas intoxication.

## PATHOPHYSIOLOGY OF SULFUR MUSTARD ON SKIN

### Clinical and physiological characteristics

**Figure 1:**
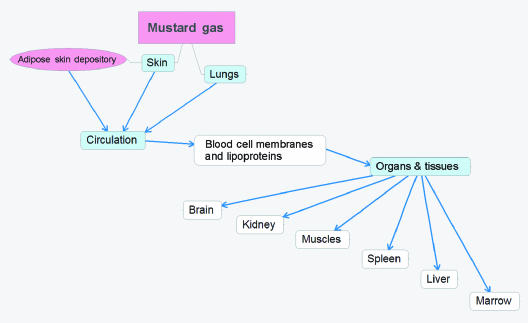
Distribution and accumulation of HD via circulation after dermal/inhalation exposure.

Mustard gas is lethal in high doses and causes severe damage to the interface organs, that is, skin, lungs, respiratory tract, and eyes. The most prominent toxic effects of HD are on skin where it produces severe damage including extremely slow healing lesions and blisters which can ulcerate, vesicate, and promote secondary infections. Because of its hydrophobic nature, mustard gas easily penetrates and accumulates in the lipid component of exposed tissues. Upon contact with the skin, about 80% of HD evaporates and only about 20% is absorbed by the skin. Skin not only accumulates but also distributes HD to other tissues. Only about 10%–12% of the initially absorbed HD is retained in the skin, whereas up to 90% of HD enters circulation as indicated in Figure 1,^1^ Extractable skin reservoirs of HD can be found in the dermis and epidermis even 24 to 48 hours postexposure.^2^ In the case of a lethal poisoning, HD concentration in skin blisters remains very high even 7 days after exposure.^3^ Consequently, even after the initial exposure, skin reservoirs continue to distribute HD via circulation to the body tissues thereby increasing damage to several organs. Figure 1 schematically shows the distribution pathway of HD toxicity throughout the human body. We would like to point that, although skin is the initial accumulator of HD, its toxic effect is also prominent in distal organs. Therefore, the effect of HD after dermal exposure is not limited only to skin tissues

While the epidermis contains no blood vessels, both the dermis and the subcutaneous regions are rich in blood vessels. Adipose cells in the subcutaneous skin layer are likely to be a depository for HD due to their high lipid content (as indicated in Figure 1). Moreover, HD solublized in adipose cells would be out of contact with water and thereby resistant to hydrolysis. After acute skin exposure, HD would be systemically delivered to various tissues in the body via lipid rich blood cell membranes and plasma lipoproteins and accumulate in lipid rich tissues (adipose tissues, brain, and skin). Chemical analyses following acute HD exposure show a high accumulation in thigh fat, brain, abdominal skin, kidney, and muscle tissues, in decreasing order.^3^ In addition, HD can be found in the spleen, liver, and bone marrow.^4^ The organs acquiring the most damage after dermal and/or respiratory exposure are indicated in Figure 1.

Skin damage caused by aerosolized HD appears after a latent period of up to 24 hours. First symptoms, such as itching, burning, and erythema, are followed by hyperpigmentation, tissue necrosis, and blister formation in warm moist areas of the body. When a large skin area is exposed to HD, medical conditions can be complicated by fluid imbalance, general inflammation, systemic intoxication, and secondary infection. At high doses, HD can also produce systemic effects with gastrointestinal symptoms (nausea and vomiting), respiratory distress due to the bronchospasm, temporary blindness as well as corneal damage. In most lethal cases, massive skin burns and wounds, as well as lung damage, are the primary causes of death. Since it damages DNA, mustard gas promotes mutagenesis and carcinogenesis.^1^, ^5^–^7^ Acute and severe exposures to HD have been shown to produce skin cancers.^8^, ^9^

A few limited cases of HD exposure in humans provide some evidence for oxidative stress. HD metabolites derived from hydrolysis (thiodiglycol, thiodiglycol sulphoxide), as well as HD metabolites from glutathione (GSH) conjugates by the beta-lyase pathway, can be found in human urine after HD exposure.^10^, ^11^ Both thiodiglycol sulphoxide and beta-lyase metabolites can be detected indicating GSH conjugation. Thiodiglycol has also been detected in urine samples from individuals not exposed to HD and is therefore not useful as a definitive marker for HD exposure.^11^ In contrast, HD metabolites from the glutathione (GSH)/beta-lyase pathway are specific for HD exposure.^10^, ^11^ These observations suggest that GSH depletion occurs in humans, and that GSH-HD/beta-lyase pathway metabolites provide a specific and useful biomarker for diagnosing HD exposure. GSH is a key intracellular antioxidant and its depletion by HD would be expected to increase oxidative stress.

**Figure 2:**
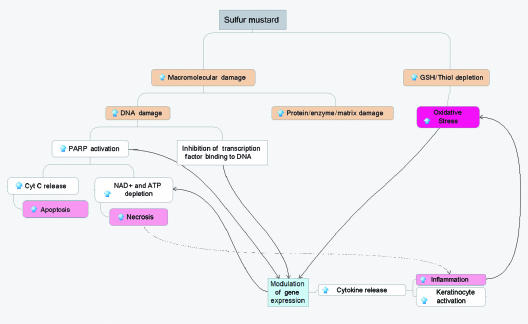
Schematic representation of the hypothesized molecular mechanisms of HD toxicity in skin cells.

The effects of HD in humans are very complicated and not fully elucidated. Figure 2 summarizes some of the key potential molecular mechanisms for HD toxicity in skin cells (as discussed in more detail below). Macromolecular damage and thiol depletion are primary and presumably the most dangerous intracellular events following HD exposure to skin cells. Macromolecular damage includes DNA damage as well as covalent modification of proteins and inactivation of enzymes. HD can affect cellular proteins indirectly by influencing expression and directly by altering the function of various enzymes and causing fragmentation of the extracellular matrix and cell detachment. GSH and total cellular thiol depletion is considered to be the major source of the oxidative stress. These primary damaging events modulate gene expression and induce inflammation and oxidative stress, which finally leads to apoptosis and/or necrosis (see Fig 2).

## GENERAL COUNTERMEASURES

Presently, elimination of contact, decontamination, and supportive therapies are the only primary treatments for vesicant exposure. Respirators and protective masks are effective in preventing inhalation, and special protective clothing can be used to eliminate skin exposure. Various decontaminating agents can eliminate or effectively reduce the toxic effect of HD if used immediately after the exposure. Ambergard XE-555 Resin reactive powder, hypochlorite neutralizing solutions, reactive skin lotions, and absorbent powders can be used to remove HD from human skin. Substantial HD reservoirs can be found in human skin even 24 hours after exposure.^12^ These reservoirs can account for up to 35% of the total dose, and it is important, therefore, to develop decontaminating techniques capable of the removing such reservoirs thereby reducing further skin and systemic damage. Graham et al^13^ have provided an excellent review of the strategies and current therapies for treating cutaneous HD toxicity and promoting wound healing. An optimal therapeutic approach is, however, still lacking.

In this review, we will focus on the potential role of antioxidant therapy; we will review the data from in vivo and in vitro models suggesting that oxidative stress is an important molecular mechanism underlying HD toxicity and that antioxidants can be therapeutically useful. The strengths and limitations of the in vivo and in vitro models will be detailed.

## MUSTARD GAS/ANALOG-INDUCED OXIDATIVE STRESS IN ANIMAL MODELS AND THE EFFECTS OF ANTIOXIDANTS

Detailed information about HD toxicity to human skin, especially at the molecular level, is very limited. Animal models are, therefore, the major source of information about the pharmacokinetics and the molecular mechanisms of HD skin toxicity. Unfortunately, there is no animal model that exactly mimics the development of HD injury in human skin. Young swine and miniature swine skin are, however, considered to be the best models since they have a similar skin structure (epidermis, dermis, and subcutaneous tissue) and barrier function. Furred animals are poor models probably because their skin is not as well keratinized as human skin, thereby permitting more rapid penetration of drugs or toxins.

Despite limitations, the mouse ear model, the rabbit, the hairless guinea pig, the nude mouse, and the weanling swine have all been useful for studying the (1) pathophysiology, (2) molecular mechanism of action, and (3) efficacy of countermeasures for HD injury. Studies on the Yucatan mini-pig have demonstrated that laminin in the dermo-epithelial junction is a target for partial protease degradation following HD exposure. The protease cleavage of laminin networks may account for the blistering effect of HD.^14^ The logistics of dealing with even miniature swine has, however, limited their use in HD studies.

In 2002, Naghii^15^ reviewed much of the existing literature connecting HD toxicity and oxidative stress and suggested that further studies in animal models were well justified. Direct evidence for free radical formation in rat lung lavage following inhalation of HD vapor has been obtained by using electron paramagnetic resonance (EPR) and spin trapping techniques.^16^ These studies show a rapid formation of ascorbyl radicals followed by the formation of carbon-centered radicals.^16^

Elsayed et al^17^, ^18^ have demonstrated that subcutaneous injections of either a butyl 2-chloroethyl sulfide (a monofunctional mustard analog) or 2-chloroethyl 4-chlorobutyl sulfide (a bifunctional mustard gas analog) in animal models caused an elevation in lung tissue lipid peroxidation as assayed by the thiobarbituric acid (TBA) assay. The TBA assay is, however, not very specific: rather then directly measuring levels of lipid hydroperoxide, this assay is generally considered a measure of total “thiobarbituric acid reactive substances” (TBARS). Total (GSH+GSSG) and oxidized (GSSG) glutathione contents in lung tissue were found to increase 1 hour and 24 hours after subcutaneous injection of butyl 2-chloroethyl sulfide.^18^ Subcutaneous injection of 2-chloroethyl 4-chlorobutyl sulfide was associated with increased GSSG and decreased GSH at 1 hour postexposure. The increased formation of GSSG and TBARS in lung tissues following subcutaneous injection of mustard analogs is consistent with oxidative stress and suggests that dermal exposure can impact distal organs. This notion is supported by the work of Vijayaraghavan et al,^19^ who found that dermally applied HD induces hepatic lipid peroxidation and GSH depletion in mice. In this study, the generation of malondialdehyde (MDA) was used as an indirect measure of lipid peroxidation Vitamin E or flavonoids, while not influencing hepatic GSH depletion, did reduce MDA levels, suggesting a therapeutic potential.^19^

The effects of topically applied HD on key antioxidant enzymes have been measured but with conflicting results. For example, Husain et al^20^ found that HD decreased the levels of glutathione peroxidase in white blood cells, spleen, and liver compared to control. Elsayed et al,^18^ however, found an increased level of glutathione peroxidase compared to controls. Elsayed^17^ interpreted the increased level of glutathione peroxidase (and other antioxidant enzymes) as an upregulation in response to oxidative stress, whereas Husain et al^20^ interpreted the decreased levels of glutathione peroxidase as a potential cause of oxidative stress. Careful in vitro work with purified enzymes may help clarify these issues.

Despite the importance of skin itself as a primary target for HD toxicity, this organ has not been extensively studied with respect to oxidative stress. Yourick et al,^21^ using the hairless guinea pig model, analyzed the skin NAD+ and NADP+ content as a function of time after HD exposure. Skin NAD+ content was found to decrease to a minimum after 16 hours (20% of control) whereas NADP+ levels increased (260%) between 1 and 2 hours and returned to control levels at 4 hours. This marked increase in NADP+ levels was thought to be an early marker of oxidative stress and a contributory factor for HD toxicity.^21^ Increased NADP+ levels are a result of increased NADPH consumption: NADPH is a major source of reducing equivalents for key antioxidant enzymes such as glutathione reductase/peroxidase and thioredoxine reductase/peroxidase and lack of NADPH would be a source of oxidative stress.

The data presented above support the view that diminished antioxidant protective mechanisms are a consequence of HD exposure. It is less clear, however, whether or not the resulting oxidative stress is a direct contributing factor to mustard toxicity or a secondary effect due to inflammation. In any event, the ability of exogenous antioxidants (as discussed below) to decrease HD toxicity supports the hypothesis that decreasing oxidative stress and/or inflammation is a viable therapeutic strategy.

### Antioxidant protection in animal models

As early as 1985, work by Vojvodic et al^22^ demonstrated that vitamin E was very effective in extending the survival time of rats acutely poisoned by HD. Vitamin E is, however, a generic term referring to at least 4 different tocopherols (alpha-, beta-, gamma-, and delta-) and 4 tocotrienols (alpha-, beta-, gamma-, and delta-). The particular form of vitamin E used in the Vojvodic et al experiments was not specified.^22^ Vitamin E is generally considered to be the primary lipid soluble antioxidant but it is now recognized that vitamin E has important “nonantioxidant” roles in modulating various signal transduction and gene regulation pathways.^23^–^25^ Moreover, the different chemical forms of vitamin E are now known to have distinct chemical and biological properties.^26^, ^27^ It is important, therefore, to specify the particular chemical and stereochemical form of vitamin E used in a given experiment.

Superoxide dismutase (SOD, EC 1.15.1.1) is a key antioxidant enzyme that catalyzes the dismutation of superoxide radicals into oxygen and hydrogen peroxide. Eldad et al^28^ studied the therapeutic role of both Cu-Zn-SOD (cytosolic form) and Mn-SOD (mitochondrial form) in HD skin damage, using the Hartley guinea pig model. Pretreatment of the animals by intraperitoneal injection with either form of SOD resulted in a dramatically reduced skin lesion area induced by HD.^28^ Treatment with SOD was, however, not effective when given 1 hour after HD poisoning.^28^ These data strongly suggest that superoxide radicals play a key role in HD-induced skin toxicity. Superoxide radicals alone are not a particularly damaging form of free radicals but they rapidly react with nitric oxide radicals to form peroxynitrite, which is a potent oxidant capable of causing tissue damage.^29^–^31^

HD and its analogs are alkylating agents that chemically react with and deplete biological thiols such as GSH, which is a key intracellular antioxidant. By promoting ROS generation and lipid peroxidation (as discussed above), HD will also promote the consumption of GSH and a reduced level of NADPH (see above) will inhibit the regeneration of GSH from GSSG. It is reasonable, therefore, that exogenous GSH or *N*-acetyl-L-cysteine (NAC) would help minimize oxidative stress induced by HD or its analogs. Kumar et al^32^ tested the potential protective effect of GSH given to Swiss albino female mice following acute exposure to HD by either inhalation or percutaneous routes. GSH was administered by intraperitoneal injection and the dose was 400 mg/kg of body weight, which translates into about a 20 mM concentration in blood. Survival time following inhalation exposure to HD was increased by GSH administration as well as 2 other antioxidants: trolox (6-hydroxy-2,5,7,8-tetramethylchroman-2-carboxylic acid), which is a water-soluble derivative of alpha-tocopherol, and quercetin, which a flavonoid.^32^ Inhalation exposure to HD depleted hepatic GSH levels, and increased hepatic and lung lipid peroxidation (as indirectly measured by MDA levels), and exogenous GSH was able to reduce lung and hepatic lipid peroxidation as well as prevent GSH depletion in these tissues.^32^ None of the 3 antioxidants tested were able to significantly increase survival time following percutaneous exposure to HD but exogenous GSH was effective in preventing GSH depletion in blood and liver. Surprisingly, lung levels of GSH were not altered by percutaneous HD exposure.^32^ The data present in work by Kumar et al^32^ show that the potential effectiveness of antioxidant therapy is dependent on the route of HD exposure.

The role of GSH and NAC (and other antioxidants) in attenuating acute lung injury by 2-chloroethyl ethyl sulfide (CEES) has recently been studied in a rat model in which lung damage was quantitatively measured by the extravasation of^125^-I-bovine serum albumin into the extravascular compartment.^33^, ^34^ CEES is a monofunctional analog of HD that has proven very useful in mimicking HD exposure. When the experimental animals were depleted of either complement or neutrophils prior to CEES exposure (by intrapulmonary injection) lung damage was significantly decreased.^34^ Neutrophil depletion was accomplished by IP injection of rabbit anti-serum to rat polymorphonuclear neutrophils and complement depletion by IP injections cobra venom factor.^34^ Antioxidants such as catalase, dimethyl sulfoxide, dimethyl urea, resveratrol, and NAC all provided significant protection in this animal model.^34^ NAC (an acetylated form of L-cysteine) can directly function as free radical scavenger and its metabolites are capable of stimulating GSH synthesis.^35^ NAC was found to be the most effective antioxidant among those tested^33^, ^34^ and was effective even when given up to 90 minutes after lung exposure to CEES.

In the work of McClintock et al,^33^ NAC was superior to GSH. In vitro work by Gross et al^36^ found that pretreatment of human peripheral blood lymphocytes (PBL) with 10 mM NAC elevated GSH level to 122% of untreated control but caused only a partial protective effect on HD-induced cytotoxicity. These researches also noted work by Meister and Anderson, suggesting^37^ that exogenously added GSH does not appear to enter the cell very effectively. This may help explain why NAC is superior to GSH in the work by McClintock et al.^33^

Bhat et al^38^ have studied the potential therapeutic use of lipoic acid to decrease oxidative stress and mustard gas toxicity in a rat model. Lipoic acid is a disulphide derivative of octanoic acid, and it is known to be a crucial prosthetic group for various cellular enzymatic complexes. Lipoic acid has been identified as a potent antioxidant and a potential therapeutic agent for the prevention or treatment of pathological conditions mediated via oxidative stress, as in the case of ischemia-reperfusion injury, diabetes, radiation injury, and oxidative damage of the central nervous system.^39^–^43^ Lipoic acid is taken up and reduced by cells to dihydrolipoate, a more powerful antioxidant than the parent compound, which is also exported to the extracellular medium; hence, protection is affected in both extracellular and intracellular environments. Both lipoic acid and dihydrolipoate, in addition to their direct antioxidant properties, have been shown to regenerate, through redox cycling, other antioxidants such as vitamin C and vitamin E, and to raise intracellular glutathione levels.^44^, ^45^ Bhat et al^38^ found that lipoic acid pretreatment decreased the levels of lipid peroxidation (measured as MDA) in lung, skin, and eyes in HD treated rats but was not effective posttreatment.

### Antioxidant liposomes as a potential countermeasure

Antioxidant liposomes may represent an optimal means of treating HD-induced skin lesions. The authors' laboratory is currently testing this hypothesis. The term “antioxidant liposome” is relatively new and refers to liposomes containing lipid soluble chemical antioxidants, water-soluble chemical antioxidants, enzymatic antioxidants, or combinations of these various antioxidants. Antioxidant liposomes hold great promise in the treatment of many diseases and conditions in which oxidative stress plays a prominent role.^46^, ^47^ The relative ease of incorporating hydrophilic and lipophilic therapeutic agents into liposomes; the possibility of directly delivering liposomes to an accessible body site; and the relative nonimmunogenicity and low toxicity of liposomes have rendered this system highly attractive for drug delivery. Moreover, several studies have clearly indicated that the liposomal antioxidant formulations, compared to that of the free nonencapsulated antioxidants, exert a far superior protective effect against oxidative stress-induced tissue injuries.^48^

Experimental studies have shown that liposomes and their constituents effectively penetrate skin.^49^, ^50^ Topical application of antioxidant-liposomes is likely, therefore, to be particularly effective in enhancing the antioxidant status of skin. Work by Kirjavainen et al^49^ suggests that liposomes containing dioleylphosphatidyl ethanolamine (DOPE) are better able to penetrate into the stratum corneum than liposomes without DOPE. Similarly, ultradeformable liposomes, lipid vesicles with special membrane flexibility due to incorporation of an edge activator such as sodium cholate, have been shown to be superior in comparison to ordinary phosphatidylcholine liposomes (see http://www.skin-forum.org.uk/abstracts/ebtassam-essa.php).

At present there are no data on the potential use of antioxidant liposomes in treating HD-induced skin lesions but McClintock et al^33^ have found that liposomes containing pegylated (PEG) catalase, PEG-SOD, or the combination were very effective in reducing CEES-induced lung injury in a rat model. Similarly, liposomes containing NAC, GSH, or resveratrol also were effective according to this study.

### In vitro studies using human skin models

#### Keratinocyte cell lines

In vivo models are essential for testing countermeasures to HD or its analogs, however, in vitro models are also critical for rapid screening of potential therapeutic agents and for detailed studies at the molecular level. Skin is the largest organ of the human body with a complicated multilayer multicell type structure. As mentioned above, there is no model system perfectly mimicking human skin. Normal or immortalized human keratinocytes cultured on plastic as a monolayer represent the simplest and least inexpensive model and are suitable for an initial approach for HD toxicity studies. Normal human epidermal keratinocytes (NHEK) isolated from adult or infant fetal skin tissue are available commercially. These cells are easy to handle, can be frozen for long-term storage but require special medium containing a mixture of growth factors.^51^ Even then, NHEK cells spontaneously transform after 3–5 passages as they continuously undergo terminal differentiation.

Nevertheless, NHEK remains the only commercially available normal cell line possessing all of the structural and functional features of normal skin keratinocytes and is being used by many investigators to study mustard gas toxicity.^52^ However, the requirement of special growth medium and a short lifespan make this model more expensive than immortalized human keratinocytes such as human pappiloma virus (HPV)–immortalized cell lines or spontaneously immortalized HaCaT cells. There are also a number of commercially available human keratinocyte cell lines immortalized via transfection with DNA coding E6 and/or E7 viral oncoproteins. All of these cell lines still require special medium with growth factors and, like NHEK, have a limited lifespan since they spontaneously transform after 10 to 15 passages.^53^

The HaCaT cell line, originating in Germany, has recently become commercially available; it represents spontaneously immortalized adult human keratinocytes.^54^ HaCaT cells are extremely easy to handle and do not require special medium. Theoretically, HaCaT cells have an unlimited lifespan but they do show morphological changes after 10 to 20 passages. Despite the altered growth potential, HaCaT cells still express differentiation-specific markers^54^ and unlike HPV-immortalized cell lines, HaCaT cells are not tumorigenic when transplanted into nude mice.^54^

It is well known that HD, like UV radiation, affects mostly proliferating keratinocytes within the lower dermis and basement membrane. Differentiating keratinocytes of the epidermis are much less susceptible to toxicity since they do not undergo apoptosis and respond weakly to inflammatory stimuli. Normal keratinocytes undergo terminal differentiation (so-called “cornification”) in response to a high (1 mM) exogenous Ca^++^ concentration.^51^ Normal keratinocytes in vivo start to differentiate when they detach from the basement membrane and migrate to the suprabasal layers.^55^ Thus, NHEK and HPV-immortalized keratinocytes, unlike HaCaT cells, spontaneously differentiate when subcultured in response to the cell detachment. Therefore, only the first passages of NHEK cells are truly proliferating, whereas every passage of HaCaT culture consists of proliferating cells. On the other hand, HaCaT cells show impaired production and release of IL-1^56^ which is crucial for normal keratinocyte proliferation and also plays an important role in keratinocyte activation and keratinocyte/fibroblast crosstalk in normal skin.^57^

As previously acknowledged, HD-induced depletion of intracellular glutathione (GSH) is a triggering event for oxidative stress in skin. Smith et al^58^ have shown that pretreatment of the human keratinocyte cell line, SVK-14, with GSH markedly increases the resistance to HD-induced cytotoxicity. Conversely, pretreatment with buthionine sulfoximine (BSO) increases the sensitivity of G361, SVK14, HaCaT, and NCTC 2544 human keratinocytes to HD toxicity.^59^ BSO lowers intracellular GSH by irreversibly inhibiting the rate-limiting GSH synthesis enzyme γ-glutamylcysteine synthetase. Surprisingly, there is no reported direct evidence to date for the enhanced generation of ROS and/or RNOS in HD-treated keratinocytes.

As pointed out earlier, HD and its chemical analogs cause massive leukocyte infiltration in animal skin and lungs^60^, ^61^ It is likely that lymphocytes and macrophages, attracted to the burned area by cytokines released from keratinocytes/fibroblasts, could be a major source of oxidative stress to skin cells. It has been demonstrated that HD-exposed NHEK cells express chemoattractants and cytokine.^62^–^64^ Moreover, an enhanced ability of NHEK cells to attract lymphocytes in vitro was demonstrated in an experiment in which the media from HD-treated keratinocytes was tested for chemoattractant activity to polymorphonuclear leukocytes purified from human blood.^65^

#### Multilayer keratinocyte tissues

Multilayer skin tissues (so-called “3D skin models”) are a more realistic model for toxicological studies. The simplest models of this class consist only of keratinocytes such as the commercially available Epiderm, which is a few millimeters thick structure of human NHEK cells grown on top of a wet membrane. Epiderm provides the possibility of applying HD (or other gaseous agents) in vapor or aerosol form which closely simulates a real HD attack. However, this model represents differentiating keratinocytes on a collagen matrix and practically all of the cells within the tissue start to cornify at the moment they are fully grown. Blaha et al^66^–^68^ have characterized the ultrastructural, histological, and molecular response of the Epiderm model to CEES. The Epiderm system not only has great potential for identifying and developing sulfur mustard therapeutic agents but also has limitations. In vivo, skin damage would be accompanied by the rapid leakage of serum, leukocyte infiltration, and perhaps mast cell degranulation (see below) but these events will not occur in any of the available in vitro skin models.

More advanced tissue models, like EpidermFT full thickness skin tissue model, consist of 2 cell types: a bottom layer of human fibroblasts imbedded in gelatin and an upper multilayer of human keratinocytes. This particular model is particularly valuable for studies involving paracrine signaling (keratinocyte/fibroblast interactions). HaCaT cells, with normal human or mouse fibroblasts, have also been used to construct 3D models of human skin. However, the impaired IL-1 production in these cells presents some technical difficulties that can be overcome with the addition of human growth factors.^56^ These multilayer skin models morphologically mimic the dermis and epidermis of human skin including the cuboidal appearance of the basal cell layer, the presence of the stratum spinosum and stratum granulosum with typical stellate-shaped keratohyalin granules, and the presence of numerous lamellar bodies that are extruded at the stratum granulosum–stratum corneum interface.

In a key experiment, Blaha et al^67^ compared the effects of CEES on the secretion of key inflammatory mediators using 2 model human skin systems, the Epiderm system (from MatTek Corporation) and the Skin2 system (a 3D skin model) from Advanced Tissue Sciences, which consists of differentiating keratinocytes on a fibroblast-collagen matrix. In the Skin2 system, the proinflammatory cytokine IL-1alpha increased in response to CEES but the proinflammatory cytokine IL-6 decreased: the Epiderm showed undetectable levels of IL-6 and the levels of IL-1alpha did not change in response to CEES.^67^ These data show that the presence of fibroblasts in the Skin2 model dramatically changes the cytokine secretion response to CEES.

More recently, Hayden et al^69^ evaluated the effects of HD on the EpiDermFT skin model which has a 3D, highly differentiated human skin-like structure with an epidermis and a dermis. This in vitro model permits the study of dermal phenomena in which fibroblast-keratinocyte cell interactions are important as appears to be the case for CEES-induced skin injury (see above). Hayden et al^69^ treated the EpiDerm-FT model with HD for 8 minutes and evaluated the structural effects at 6 and 12 hours postexposure. Histological analyses showed typical HD targeting of basal keratinocytes (cytopathology, condensed chromatin, pyknotic nuclei, and increased eosinophilia) and epidermal cleavage at the dermal/epidermal junction. Transmission electron microscopy showed that lamina densa of the basement membrane to be largely intact. The EpiDerm-FT model represents a major advance in the development of human skin models and its use in studying the molecular mechanisms/proteomics for HD/CEES toxicity is just beginning to be exploited.

#### Human skin allografts in immunodeficient mice

A third class of human skin model is provided by the use of human skin allografts in immunodeficient mice. Human skin cells, either genetically modified^70^ or normal,^71^ were grafted onto nude mice and successfully used to examine HD-induced biochemical alterations in skin. In 1995, Rosenthal et al^70^ described an engineered human skin model, in which human keratinocyte clones, with some genetic modifications, were grafted onto nude mice, where they formed histologically normal human skin. Later, the same group reported an advanced model developed in immunodeficient nude mice, where a pellet of cells containing human keratinocytes and fibroblasts were placed on top of the muscular layer at the graft site and grown for 1 week.^71^ Glass bulbs filled with HD can be directly applied to the sections of mouse skin containing the human skin allograft. Although these in vivo models are expensive and complicated, they possess a number of advantages over any of the in vitro cultured skin models. Grafted human skin models make it possible to obtain a detailed picture of HD-induced morphological, ultrastructural, and inflammatory alterations in various layers of skin cells possessing the realistic complexity of multiple cell–cell interactions. Recently, 3D human skin allografts in mice have allowed investigators to identify distinctive prevesication and postvesication phases and to monitor both dermal-epidermal separation and basal membrane alterations in response to HD exposure.^72^, ^73^ However, a limitation of this model is the lack of a functional immune response in the recipient mice.

In spite of the ever higher degrees of physiological complexity, there is not a single model that reflects all the features of human skin. The choice of a particular model may, therefore, be dictated by the particular experimental design and goals. Wound healing studies, for example, would require an in vivo system with an intact immune system since immune cells are known to contribute to skin regeneration. Moreover, immune cells are thought to play important roles both in HD-induced skin inflammation and in postexposure wound healing through the expression of proinflammatory cytokines (such as Il-1β, TNF-α, IL-6, and GM-CSF) within the first hour after exposure and proceeding through vesication and blister formation.^64^ Leukocyte infiltration always starts shortly after HD treatment in mice, rabbits, or guinea pigs. In wound healing, leukocytes and macrophages provide many of the molecular signals regulating fibroblast and keratinocyte proliferation.

The effects of HD on all of the various cell types that come in contact with skin, including macrophages and mast cells, are also important in understanding the overall effects of HD on skin in vivo. Rikimaru et al^74^ have used full-thickness human skin explants to study inflammatory mediators in response to topically applied HD. These investigators found that culture fluids from the HD-treated skin contained increased levels of histamine, plasminogen-activating activity, and prostaglandin E2 compared to control explants. It was concluded that both mast cells and epidermal cells were apparently involved in early mediation of the inflammatory response to HD.^74^ In contrast, Inoue et al^75^ found that the inflammatory response of the mouse ear to HD did not differ in mast cell deficient mice compared to normal mice. At present, there is no obvious explanation for the differences observed between the work of Rikimaru et al^74^ and that of Inoue et al^75^ It may well be that the mouse ear is not an optimal model for human skin. It is critically important to determine whether HD, or other toxic vesicants, degranulate mast cells since this process could be a major source of inflammatory mediators and, therefore, a major factor in modulating the immune response to HD. In particular, mast cell degranulation would release large amounts of TNF-alpha, which is an inflammatory cytokine.

**Figure 3:**
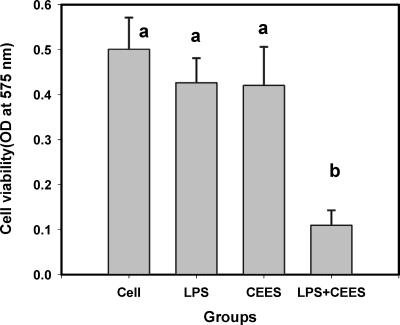
LPS (100 ng/mL) enhances the cytotoxicity of CEES (500 μM). Means not sharing a common letter are significantly different (*P* < .05). Cytotoxicity was measured after 24 hours by the MTT assay.

**Figure 4:**
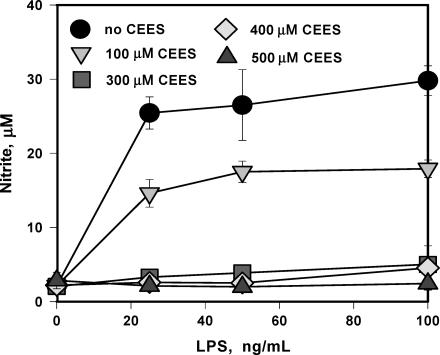
CEES inhibits NO production in LPS stimulated RAW 264.7 macrophages. Cells were simultaneously treated with various levels of CEES (as indicated) and low doses of LPS (as indicated). NO production was monitored as the concentration of nitrite in the culture media after 24 hours.

We have previously reported that lipopolysaccharide (LPS) as well as other inflammatory factors such as TNF-alpha and IL-1-beta amplify the toxicity of CEES^76^ and that CEES is a potent inhibitor of nitric oxide production from inducible nitric oxide synthase (iNOS).^77^ LPS is a major component of the cell wall of gram-negative bacteria and is known to trigger a variety of inflammatory reactions in macrophages and other cells having CD14 receptors.^78^, ^79^ In particular, LPS is known to stimulate the macrophage secretion of nitric oxide^80^ and inflammatory cytokines such as tumor TNF-alpha and IL-1-beta^81^ Figure 3 shows that RAW 264.7 macrophages stimulated with LPS at 100 ng/mL are markedly more susceptible (*P* < .05) to CEES cytotoxicity (24 hours with 500 μM) than resting macrophages as indicated by a dramatic drop in dehydrogenase activity as measured by the MTT ((3-(4,5-dimethylthiazol-2-yl)-2,5-diphenyltetrazolium bromide) assay. In the absence of LPS, CEES at a level of 500 μM did not significantly affect cell viability.^76^ Figure 4 shows that CEES (100–500 μM for 24 hours) inhibits the secretion of nitric oxide into the cell medium by LPS stimulated macrophages in a dose-dependent manner^77^ In these experiments, nitrite secretion into the cell culture medium was used as a measure of nitric oxide synthesis. Macrophages (and mast cells) are both present in dermal tissues.

IgE-mediated mast cell degranulation is known to be inhibited by nitric oxide production.^82^ NO is a powerful antioxidant 83 and increased intracellular levels of NO are known to inhibit mast cell degranulation.^84^ Significantly, mast cell degranulation and histamine release are stimulated by membrane lipid peroxidation and inhibited by antioxidants such as alpha-tocopherol.^85^ Collectively, the information presented above suggests that HD/CEES could induce mast cell degranulation by increasing oxidative stress and/or decreasing nitric oxide production. The subsequent release of TNF-alpha could enhance the cellular toxicity of HD/CEES.

NO generation, mediated by iNOS, is also crucial for the rapid healing of human skin wounds. Although, keratinocytes are known to express iNOS and generate NO in wound healing, it is likely that macrophages, known for their ability to express iNOS and generate high levels of NO, also contribute to the healing stimuli. Thus, it is tempting to suggest that the development of a 3-cell-type (macrophages/fibroblasts/keratinocytes) model would provide a unique and optimal model for studying skin vesication, blistering, and wound healing under very reproducible experimental conditions.

## MOLECULAR MECHANISMS FOR MUSTARD TOXICITY

The molecular mechanisms of HD skin toxicity are complex and not yet fully understood. We will focus on 3 major types of interrelated events: primary macromolecule damage, oxidative stress, and inflammation. All of these processes are tightly interconnected and play central roles in HD toxicity. The hypothesized mechanisms of HD toxic effect in skin cells are summarized in Figure 2. In addition, we will discuss the importance of NO signaling modulation in HD toxicity since it is likely to be important for the postexposure wound healing process in skin.

### Primary macromolecule damage

HD easily penetrates both cellular and nuclear membranes due to its hydrophobic nature. In the cytosol, it reacts with water forming a highly electrophilic ethylene episulfonium derivative that is the ultimate alkylating agent. DNA alkylation and crosslinking are well-documented primary intracellular damaging reactions of HD. Extensive DNA damage, due to alkylating agents, activates and overloads the DNA repair machinery. In particular, DNA damage induces expression of poly (ADP-ribose) polymerase (PARP), the key regulatory enzyme involved in DNA repair and hypothesized to regulate cell fate by modulating death and survival transcriptional programs.^86^ HD induces PARP expression in normal human keratinocytes and the possible involvement of this nuclear enzyme in the regulation of HD cell death mechanisms has been extensively studied.^87^–^91^

PARP-1 is the most abundant member of the of PARP protein family. PARP-1 binds to DNA structures that have single- and double-strand breaks, crossovers, cruciforms, or supercoils^92^; it signals DNA rupture and facilitates base-excision repair.^93^, ^94^ Normally, the intracellular level of PARP-1 is very low, and this enzyme can be detected in the cytosol only under stressful conditions. Upon binding to the damaged DNA sites, PARP-1 metabolizes β-nicotinamide adenine dinucleotide (NAD^+^) into branched polymers of ADP-ribose that are transferred to a set of nuclear proteins. This process also results in a very large decrease in the pyridine nucleotide pool. Poly(ADP-ribosyl)ation is thought to be beneficial for genome repair since modifications of proteins proximal to the DNA breaks facilitate multiple local openings of the condensed chromatin structure allowing the binding of the repair protein complex.^93^, ^95^

Despite the beneficial effect, PARP can induce apoptosis or necrosis in skin cells treated with HD^91^ or other alkylating agents.^86^, ^96^ Thus, limited expression of PARP proteins helps in DNA repair and promotes cell survival but its overexpression (as in case of massive DNA damage) can induce cell death.^97^ PARP overproduction, especially in cells utilizing aerobic glycolysis, can lead to the depletion of cellular NAD^+^ and ATP (see Fig 2) which rapidly promotes general intracellular bioenergetic collapse and oxidative stress resulting in a regulated form of necrosis.^86^, ^96^, ^98^–^100^ HD is cytotoxic to both dermal fibroblasts and epidermal keratinocytes. It has been suggested that PARP determines the mode of HD-induced cell death in skin fibroblast but not in keratinocytes.^91^ In mouse skin fibroblast the absence of PARP shifts the mode of HD-induced cell death shifts from necrosis to apoptosis,^91^ whereas keratinocytes, with or without PARP, primarily express an apoptotic form of cell death.^91^ HD-treated human keratinocytes show a PARP activation, an upregulation of proapoptotic p53 accompanied by a downregulation of antiapoptotic Bcl-2, and, finally, to caspase activation and apoptosis.^87^, ^90^ This pathway was found to be Ca^++^ and calmodulin dependent.^90^

Necrosis due to PARP-induced depletion of NAD^+^ and ATP exhaustion during aerobic glycolysis is thought to be the main mechanism of cell death induced by DNA damaging agents, especially in proliferating cells.^96^ However, these observations may vary with the dose of alkylating agent, with cell type and perhaps the particular composition of the culture medium (see below) in the case of in vitro studies. HD promotes apoptosis in HeLa cells (10–100 μM),^101^ peripheral blood lymphocytes (6–300 μM),^102^ keratinocytes (50–300 μM),^53^, ^87^ and endothelial cells (<250 μM).^103^ A time-dependent shift to necrosis was observed in HD-treated lymphocytes.^102^ but a shift toward necrosis is observed at higher levels of HD in endothelial cells (>500 μM)^103^ and HeLa (1 mM)^101^ Interestingly, human fibroblasts undergo necrosis even at lower concentrations of HD (100–500 μM).^91^ In most human cell types, apoptosis predominates within 6–12 hours of postexposure time, whereas necrotic events markedly increase after 12–24 hours.

Countermeasures capable of preventing rapid ATP depletion and mitochondrial dysfunction could be protective against HD-induced necrosis. Unfortunately, this approach would not eliminate cell death completely since apoptosis would likely proceed. However, a shift from necrosis to the less inflammatory apoptotic pathway could possibly be beneficial by helping eliminate secondary infections and improving wound healing. PARP activation causes NAD^+^ depletion and NAD+ is required for glycolysis and pyruvate synthesis.^104^ In the absence of pyruvate, mitochondrial respiration fails causing bioenergetic collapse and cell death via necrosis.^86^ Therefore, the addition of a mitochondria substrate, such as pyruvate, glutamate, or glutamine, to the cell medium could be protective against necrosis.^104^ A protective effect of pyruvate treatment has, indeed, been documented in genotoxic stress caused by nitrogen mustard or *N*-methyl-*N′*-nitro-*N*-nitrosoguanidine (MNNG), a chemical analog of HD used as anticancer drugs.^96^, ^100^, ^104^

Alkyl pyruvates, such as methyl pyruvate and ethyl pyruvate, are excellent alternative mitochondrial substrates since they (unlike pyruvate) are stable in solution. In aqueous solutions, pyruvate spontaneously undergoes a series of chemical reactions yielding 2, 4-dihydroxy-2-methylglutarate, which is a mitochondrial poison.^105^ In addition, alkyl pyruvates also function as effective and potent scavengers of free radicals. Pyruvates are capable of scavenging hydrogen peroxide (H_2_O_2_) and the hydroxyl radical (OH&rad;^−^).^106^, ^107^ Administration of pyruvates was shown to protect against various types of oxidant-mediated cellular and organ injuries in numerous in vitro and in vivo studies.^108^, ^109^ These data further suggest that HD activation of PARP and the subsequent depletion of pyruvate is also a contributing factor for HD-induced oxidative stress.

In preliminary results, the authors' laboratory has observed that methyl pyruvate provides protection to human keratinocytes (HaCaT cell line) treated with CEES and a similar effect was observed with ethyl pyruvate (unpublished data). It is worth noting that commercially available serum-free media, formulated to culture NHEK cells, contains 0.5 mM sodium pyruvate. Keratinocyte media from Gibco, Sigma, and Cambrex all contain 0.5 mM pyruvate. It is possible, therefore, that necrosis has not been observed in HD-treated NHEK cells due to the protective effect of sodium pyruvate in the culture media.

In our experiments (unpublished data), however, we used immortalized HaCaT keratinocytes, which proliferate continuously but do not differentiate. Actively proliferating cells utilizes aerobic glycolysis and are more susceptible to mitochondrial dysfunction and necrosis.^86^, ^96^, ^100^ After limited number of passages NHEK cells, unlike HaCaT cells, undergo terminal differentiation which is a form of cell death different from either apoptosis or necrosis.^55^ This difference between HaCaT and NHEK cells theoretically could cause discrepancies in the cell death pathway caused by HD. Parallel experiments are now being done with NHEK and HaCaT cells to further characterize the protective effect of pyruvate to HD/CEES.

### Inflammation

HD-treated normal human keratinocytes release proinflammatory TNF-α, IL-6, IL-1β in a dose-dependent manner^62^ but the particular cytokine profiles observed differ depending on the skin model used and the dose of HD.^63^, ^64^, ^110^ Cytokine production and responses are known to be regulated by the activation of nuclear transcription factor-kappaB (NF-kappaB) and this activation also plays a key role in determining the fate of a damaged cell. There are numerous activators of NF-kappaB such as bacterial and viral infections, chemical damage, radiation, and oxidative stress. In response to these stimuli, an active NF-kappaB protein complex is liberated in the cytoplasm and it subsequently translocates to the nucleus and triggers selective gene expression. Among the genes regulated by NF-kappaB are adhesion molecules, pro-inflammatory mediators (IL-1 beta, TNF-alpha, interleukin 6), chemokines, IL-8, iNOS, E-selectin, vascular cell adhesion molecule 1 (ICAM-1), and granulocyte-macrophage colony stimulating factor (GM-CSF).^34^, ^111^–^114^ In general, NF-kappaB activation also triggers antiapoptotic genes and promotes cell survival.

Although the precise mechanism(s) of HD-induced gene expression has not yet been fully described in skin cells, it is very likely connected to the DNA damaging effect of HD (see Fig 2) and could, therefore, be PARP-dependent. PARP-1 is known to be a coactivator of NF-kappaB.^115^; however, this pathway has not been fully explored in HD-treated keratinocytes or fibroblasts. It is also possible that HD modulates NF-kappaB and other nuclear factors by covalently modifying DNA binding sequences for transcription factors. Grey et al^116^ have shown that HD inhibits the in vitro binding of transcription factor activating protein-2 (AP-2) via alkylating the AP-2 DNA consensus binding sequence rather than by direct damage to the AP-2 protein.

In addition, it is highly possible that HD-induced oxidative stress also can stimulate inflammatory responses via transcription factors. Many of the activators of NF-kappaB can be blocked with the use of antioxidants.^117^, ^118^ Transcription factors AP-1,^119^, ^120^ MAF and NRL,^121^, ^122^ and NF-IL6^123^, ^124^ are regulated by oxygen-dependent mechanisms, and sensitive to ROS. Interestingly, chemical compounds indirectly disrupting NF-kappaB activation induce apoptosis in cancer cells,^125^–^130^ whereas inhibitors of NF-kappaB activation protect HD-treated human keratinocytes.^131^, ^132^

It is also unclear how exactly the proinflammatory cytokines, such as IL-6, contribute to the HD-induced skin damage. It is known, however, that inflammatory processes contribute to the skin damage. In animal models, both skin and lung exposure to HD or CEES causes massive leukocyte infiltration, which starts shortly after the exposure and builds up continuously.^60^, ^61^ The fact that skin burns and blistering have a latent period also suggests that secondary responses in skin cells/immune cells also contribute to the mechanisms of HD toxicity. HD treatment is known to induce NF-kappaB activation and release of inflammatory cytokines in both keratinocytes and macrophages.^63^, ^110^, ^133^–^135^ TNF-alpha, in general, induces apoptosis in keratinocytes and treatment with anti-TNF-alpha antibodies is protective against UV-induced skin lesions.^136^ However, the effect of TNF-alpha in HD-treated skin is complex and an attempt to reduce cell death in normal human keratinocytes by blocking TNFR1, the major cell receptor for TNH-alphas was not successful.^71^

It is likely that keratinocyte activation (see Fig 2) plays an important role in HD toxicity. Activation of keratinocytes is a multistep pathway induced in response to skin injury. Activated keratinocytes are hyperproliferative and able to migrate to the site of injury in order to form layers of fresh cells in the dermis and epidermis. Activation is regulated by various cell signaling pathways including TNF-alpha.^57^, ^137^ Since HD-treated keratinocytes release high levels of TNF-alpha in the medium,^133^ we suggest that HD promotes keratinocyte activation, subsequent hyperproliferation, and an enhanced susceptibility to the PARP-mediated bioenergetic collapse. This series of molecular events would cause a shift from apoptosis to necrosis. Although a time- or concentration-dependent shift from apoptosis to necrosis has been well documented for HD-treated lymphocytes,^102^ endothelial cells,^103^ and HeLa cells,^101^ such changes have not been noted for human keratinocytes. As discussed above, this may be due to the fact that NHEK cells are always protected from necrosis by pyruvate-containing media. The recently documented protective effect of NF-kappaB inhibitors in NHEK and HaCaT cells treated with HD^131^, ^132^ also supports our speculation since these inhibitors downregulate TNF-alpha which would impair keratinocyte activation.

## WOUND HEALING

NO signaling plays a key role in the inflammation and wound healing.^138^–^140^ Animal studies^141^ have shown that in iNOS knockout mice, wound healing is impaired but restored by iNOS gene transfer. Lack of NO and impaired expression of iNOS after the HD exposure are thought to be important events promoting skin burns and blistering. We have shown that HD treatment inhibits iNOS expression and NO synthesis (see Fig 4) in LPS-stimulated murine macrophages.^77^ Suppression of iNOS expression and several protein activators of wound healing have also been found in human keratinocytes treated with HD.^142^

In keratinocytes, the beginning stage of the wound healing process is determined by the activation process. Under conditions of physical injury, the keratinocyte cell cycle is activated and the cells become hyperproliferative and migrate to the site of injury in response to chemokines.^57^, ^137^ Activation of keratinocytes, as well as their return to the healthy basal phenotype, is controlled by cytokines and growth factors produced by various cutaneous cell types, including keratinocytes and lymphocytes infiltrated at the wound site. Various intracellular signaling pathways are involved at the different stages of activation. Interestingly, NF-kappaB activation and consequent autocrine TNF-alpha production occur at the initial stages of the activation and allow keratinocytes to become hyperproliferative and migratory.^57^, ^137^ Activation is terminated when lymphocytes, present at the wound site, release interferon-gamma (IFN-gamma), which induces the activation of STAT-1 and makes keratinocytes contract newly deposit fibronectin-rich basement membrane. Finally, transforming growth factor-beta (TGF-beta) secreted by fibroblasts induces the expression of K5 and K14, fully reverting the keratinocytes to a healthy basal phenotype and making them responsive to differentiation stimuli.^57^

In human cells, expression of iNOS, which is the main NO-generating protein in keratinocytes,^143^ is regulated synergistically by 2 major pathways: NF-kappaB and STAT-1.^144^ As pointed above, both HD and its chemical analog CEES downregulate iNOS expression in murine macrophages^77^ and human keratinocytes.^142^ Since NF-kappaB activation is well documented in NHEK cells,^131^–^133^ it is possible that the impaired expression of iNOS in HD-treated cells could be attributed to a STAT-1-dependent mechanisms. In addition, the possible STAT-1 inhibition by HD could disrupt the IFN-gamma signaling pathway resulting in keratinocytes unable to terminate their wound healing state.^57^ Thus, simultaneous HD-induced NF-kappaB activation and STAT-1 inhibition could alter necrosis, inhibit NO generation, prevent wound healing, and possibly affect vesication and blistering.

The molecular mechanisms whereby HD alters transcription factors activation are not fully elucidated. However, it is highly possible that the ability of mustards to alkylate DNA is involved. As pointed above, HD is capable of chemically modifying both proteins (via crosslinking of Cys residues) and DNA (via alkylation of guanine rich sequences, and crosslinking). It seems likely that HD would more effectively damage “more exposed” regions of DNA with accessibility to transcription factors. Interestingly, Gray^116^ had shown that HD inhibits the in vitro binding of transcription factor AP-2 via alkylation of the guanine-rich consensus DNA sequences but not by directly damaging the AP-2 protein. It is tempting to assume that other transcription factor functions could be affected by HD in a similar manner. However, the effects of HD on NF-kappaB and STAT-1α have not been elucidated and AP-2 remains the only transcription factor studied in relation to HD toxicity.

Since some transcription factors are sensitive to ROS and to the redox state of the cell in general, it is likely that oxidative stress, inflammation, and NO signaling are tightly interconnected in skin and its dynamic responses to toxic agents. Soneja et al^145^ have suggested that wound healing could be accelerated under circumstances in which oxidative stress is minimized but NO production remains elevated. On the other hand, under conditions elevating oxidative stress, the toxicity of mustards can be greatly enhanced. For instance, the HD analog CEES shows much higher toxicity in cells stimulated by LPS, TNF-alpha, or IL-1beta, which enhance inflammation and oxidative stress.^76^

## FUTURE DIRECTIONS

### A systems biology approach to mustard toxicity

As discussed above, HD toxicity in skin results from a multistep complex mechanism involving a number of signaling cascades and various cell types. It is extremely difficult to follow each step in this mechanism even in a simple in vitro model. A systems biology approach would view HD toxicity as time-dependent disruption of an integrated and interacting network of genes, proteins, and biochemical reactions. This approach would emphasize integrating data obtained from transcriptomics, metabolomics, and proteomics with the purpose of constructing and validating a comprehensive model of HD toxicity. The computational tools for this task would include network mapping as well as correlation, logical, and kinetic modeling.^146^ This comprehensive model would be the best way to address the question: “how relevant to the HD-induced cell death pathways are the direct chemical alterations caused by HD to various cellular proteins (oxidation, cross-linking, and fragmentation) and the indirect chemical protein alterations caused by ROS and RNOS?”

A transcriptomic approach to studying HD toxicity is already yielding useful results. In NHEK cells, DNA array techniques have been applied to studying HD-altered gene expression,^147^, ^148^ and mRNA differential display has been used to examine HD-induced transcriptional modulations in human epidermal keratinocytes.^149^ Microarray analyses of gene expression in CEES- or HD-exposed mouse skin in vivo have also been accomplished.^147^, ^150^ These studies are providing a deeper insight into the mechanism of HD toxicity since they have identified a number of genes upregulated at the early (0.5–4~hours) and intermediate (24 hours) stages of postexposure. DNA array analyses are capable of providing crucial information regarding the changes in transcriptional activity in the cell and are useful in the search for “the key” signaling pathways involved in HD toxicity. These studies will help in the design of evermore effective countermeasures and help identify key biomarkers for therapeutic efficacy. Proteomic data on HD toxicity are currently very limited but this approach would complement the previously accumulated microarray data by helping identify all the key proteins involved in HD toxicity at different stages.

Collectively, the literature reviewed here supports the notion that oxidative stress, free radical damage to biomolecules, and alterations in redox sensitive signaling pathways are key factors in understanding vesicant toxicology. It is likely, therefore, that the newly emerging area of redox proteomics would be particularly useful in understanding HD damage to skin. Redox proteomics is focused on characterizing (1) the chemical modifications of specific proteins induced by ROS and RNOS; (2) alterations in specific proteins induced by changes in redox sensitive transcription factors; and (3) alterations in the function/structure of specific proteins caused by redox sensitive posttranslational modifications.^151^–^153^ In this regard, small thiols, like GSH, are no longer viewed just as protective antioxidants but as redox regulators of proteins via glutathionylation or by oxidation of protein cysteine residue.^152^ Redox proteomics is rapidly emerging as a very powerful tool for characterizing and identifying proteins based on their redox state.^153^ This approach has recently been used to specifically identify oxidized proteins in Alzheimer's disease and this information has proven useful in identifying new therapeutic targets and in providing new molecular insights into disease etiology.^154^

### Multicomponent antioxidant liposomes

HD, due to its hydrophobic nature, effectively penetrates deep into the skin and affects mostly proliferating cells within basement membrane, that is, the lowest layer of proliferating keratinocytes. These growing cells would be highly susceptible to the PARP-mediated bioenergetic collapse since they actively utilize aerobic glycolysis. HD is likely, therefore, to induce necrosis rather than apoptosis in these cells, which would subsequently promote severe inflammation, skin blistering, and vesication. Thus, it is critically important to provide fast and efficient delivery of the desired drugs to the deeper skin layers and liposomes hold promise in this regard. By encapsulating a lipid soluble thiol, antioxidant liposomes could also effectively diminish (by direct covalent reaction) the stores of HD in skin lipid depots. Although this review has emphasized antioxidants, there is practically no limit to the possible encapsulated agents that can be incorporated into liposomes and delivered to the skin cells. These agents could include PARP inhibitors, protease inhibitors, anti-inflammatory agents, and chemical or enzymatic antioxidants. Currently, we are testing novel formulations of multiagent antioxidant liposomes containing both antiapoptotic (NAC) and antinecrotic (ethyl pyruvate) agents. Liposomes can also be formulated with agents designed to accelerate wound healing such as epidermal growth factor, transforming growth factor-β, platelet-derived growth factor, insulin-like growth factor, keratinocyte growth factor, and fibroblast growth factor.

## ACKNOWLEDGMENTS

This research was supported by three United States Army Medical Research Command (USAMRMC) Grants: “The Influence of Antioxidant Liposomes on Macrophages Treated with Mustard Gas Analogues”, Grant No. 98164001; “Topical Application of Liposomal Antioxidants for Protection against CEES Induced Skin Damage”, Contract No. W81XWH-05-2-0034, and; “A Proteomic Approach for Studying the Therapeutic Use of Antioxidant Liposomes”, Contract No. W81XWH-06-2-044.
